# A web-based nomogram model for predicting the overall survival of hepatocellular carcinoma patients with external beam radiation therapy: A population study based on SEER database and a Chinese cohort

**DOI:** 10.3389/fendo.2023.1070396

**Published:** 2023-01-31

**Authors:** Gouling Zhan, Honghua Peng, Lehong Zhou, Long Jin, Xueyi Xie, Yu He, Xuan Wang, Zhangyan Du, Peiguo Cao

**Affiliations:** Department of Oncology, Third Xiangya Hospital, Central South University, Changsha, Hunan, China

**Keywords:** hepatocellular carcinoma, external beam radiation therapy, overall survival, SEER database, web-based nomogram

## Abstract

**Background:**

External beam radiation therapy (EBRT) for hepatocellular carcinoma (HCC) is rarely used in clinical practice. This study aims to develop and validate a prognostic nomogram model to predict overall survival (OS) in HCC patients treated with EBRT.

**Method:**

We extracted eligible data of HCC patients between 2004 and 2015 from the Surveillance, Epidemiology, and End Results (SEER) database. Those patients were randomly divided into a training cohort (n=1004) and an internal validation cohort (n=429), and an external validation cohort composed of a Chinese cohort (n=95). A nomogram was established based on the independent prognostic variables identified from univariate and multivariate Cox regression analyses. The effective performance of the nomogram was evaluated using the concordance index (C-index), receiver operating characteristic curve (ROC), and calibration curves. The clinical practicability was evaluated using decision curve analysis (DCA).

**Results:**

T stage, N stage, M stage, AFP, tumor size, surgery, and chemotherapy were independent prognostic risk factors that were all included in the nomogram to predict OS in HCC patients with EBRT. In the training cohort, internal validation cohort, and external validation cohort, the C-index of the prediction model was 0.728 (95% confidence interval (CI): 0.716-0.740), 0.725 (95% CI:0.701-0.750), and 0.696 (95% CI:0.629-0.763), respectively. The 6-, 12-,18- and 24- month areas under the curves (AUC) of ROC in the training cohort were 0.835 、0.823 、0.810, and 0.801, respectively; and 0.821 、0.809 、0.813 and 0.804 in the internal validation cohort, respectively; and 0.749 、0.754 、0.791 and 0.798 in the external validation cohort, respectively. The calibration curves indicated that the predicted value of the prediction model performed well. The DCA curves showed better clinical practicability. In addition, based on the nomogram, we established a web-based nomogram to predict the OS of these patients visually.

**Conclusion:**

Based on the SEER database and an independent external cohort from China, we established and validated a nomogram to predict OS in HCC patients treated with EBRT. In addition, for the first time, a web-based nomogram model can help clinicians judge the prognoses of these patients and make better clinical decisions.

## Introduction

Primary liver cancer (PLC) is the 6th most common cancer worldwide in 2020 and the 3rd leading cause of cancer-related death worldwide; hepatocellular carcinoma (HCC) is the most common type of PLC, which accounts for more than 80% of all PLC (1, 2). For HCC, treatment of early patients includes surgical resection, transplantation, radiofrequency ablation, etc. (3–5). Although surgical resection and/or liver transplantation remain the treatment of the first choice for HCC patients, most patients with HCC do not develop symptoms until intermediate and advanced stages, and only 20% of patients have a chance of surgical resection at the time of diagnosis (6).

Like surgery, external beam radiation therapy (EBRT) is one of the most common methods of treating tumors. HCC has moderate to high radiosensitivity to radiation therapy, just inferior to normal organs or tissues that are very sensitive to radiation, such as kidneys, bone marrow, lymphoid tissue, etc. Before the 1990s, due to technical limitations, large-volume liver irradiation often led to hepatotoxicity and even radiation-induced liver disease (RILD), limiting the role of EBRT in HCC patients (7). EBRT technology has undergone a series of advances in recent decades, with the application of three-dimensional conformal radiotherapy (3-DCRT), intensity-modulated radiation therapy (IMRT), and stereotactic body radiotherapy (SBRT), the accuracy of tumor lesion targeting was greatly improved while the radiation dose on the surrounding normal tissues can be substantially reduced, this significantly reduces the incidence of hepatotoxicity, so that EBRT may be one of the promising treatments for HCC patients (8–10).

In recent years, nomogram has been widely used as a prediction method in oncology, which is convenient for clinicians to use for prognosis prediction and has played an important role in promoting personalized medicine (11). As far as we know so far, there are few studies that have developed nomograms to predict prognosis in HCC patients with EBRT, and these were small studies due to the small number of HCC patients treated with EBRT (12–14). For the first time, based on the SEER database and a Chinese cohort, we established and validated such a nomogram to predict OS in HCC patients treated with EBRT. In addition, to provide patients with better medical care, we also established a web-based nomogram model that could help clinicians make better clinical decisions by judging the prognosis of these patients.

## Methods

### Data source and data extraction

**Figure 1:**
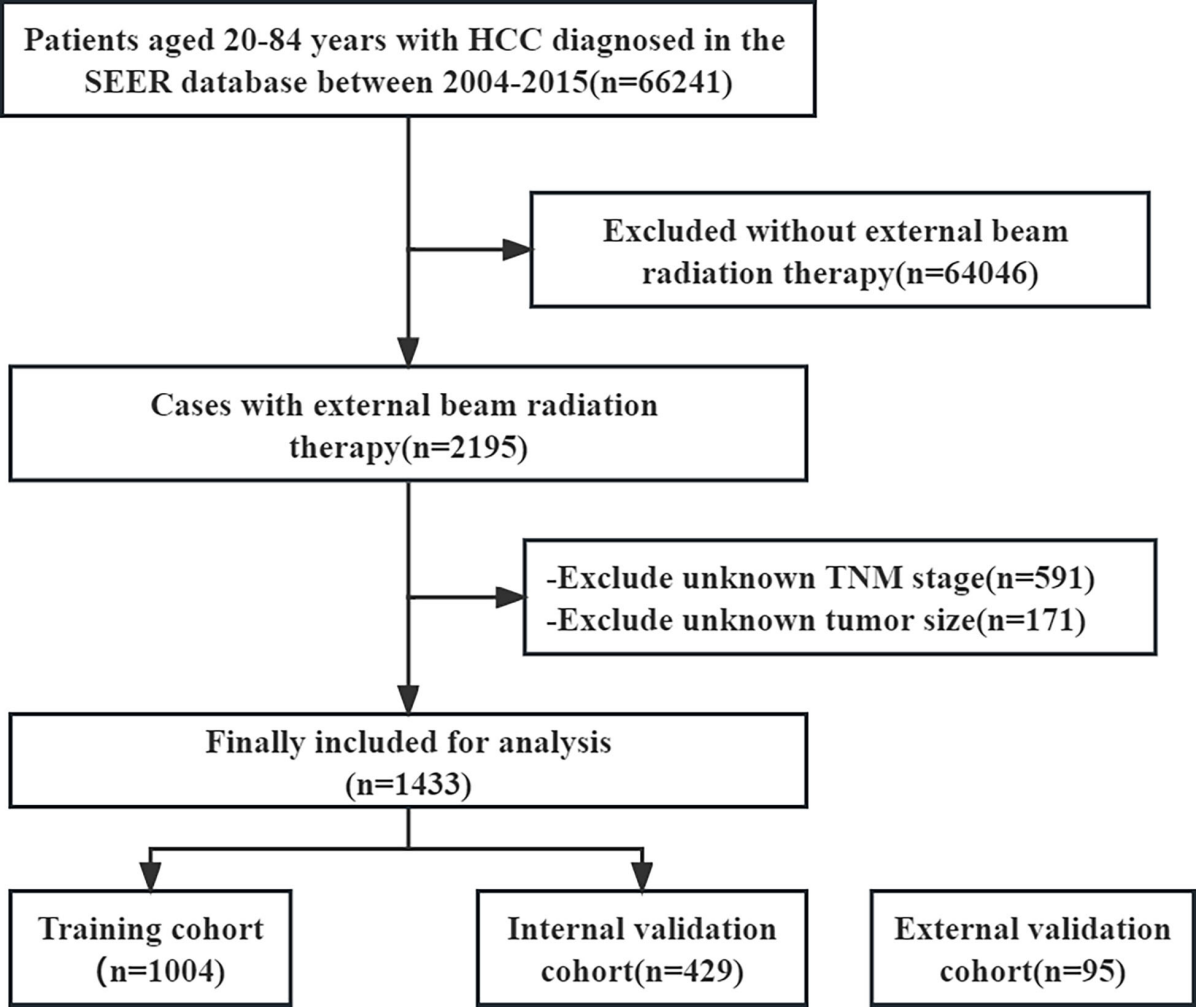
The flowchart of patient inclusion among the SEER database.

Data for related patients (from 20-84 years old) diagnosed with HCC between 2004 and 2015 were extracted from the SEER 18 registry database by SEER*Stat 8.4.0 software. The information included as following: sex, age, race, T stage, N stage, M stage, histological grade, tumor size, AFP, surgery information, radiotherapy information, chemotherapy information, survival time and vital status. Inclusion criteria included: (a) patients with HCC. Exclusion criteria included: (a) no external beam radiation therapy; (b) unknown TNM stage; (c) unknown tumor size. The flowchart for selecting HCC patients is shown in **Figure 1**.

Data of patients diagnosed with HCC between 2014 and 2021 were collected from the Department of Oncology, Third Xiangya Hospital, Central South University, Changsha, China. Inclusion criteria included: (a) HCC patients with complete clinical and pathological information; (b) Child-Pugh score liver function classifications A and B; (c) no organ function defects; (d) patients who signed the informed consent for radiotherapy and were able to comply with the treatment plan, post-treatment visits, and laboratory tests. Exclusion criteria included: (a) failure to adhere to the completion of external beam radiation therapy; (b) those with incomplete follow-up outcomes. Finally, 95 patients were included in an external validation cohort and further analysis. The study has been approved by the ethics committee of the Third Xiangya Hospital, and Individual consent was waived as a retrospective analysis.

### Statistical analysis

For the nomogram construction and validation, univariate and multivariate Cox proportional hazards regression analyses were used to identify independent prognostic factors (P<0.05) that significantly affected OS in the training cohort. We applied the Kaplan-Meier curves and log-rank test to compare patient survival between different prognostic factor groups. Using these identified prognostic factors, we constructed a nomogram for predicting 6-,12-,18- and 24- months OS rates in HCC patients with EBRT. The effective performance, predictive capacity, and discrimination of the nomogram were evaluated using the concordance index (C-index), receiver operating characteristic curve (ROC), the area under the ROC curve (AUC), and calibration curves. A decision analysis curve (DCA) is a method for evaluating the practical value of a model based on calculating the net benefit under different thresholds, and the nomogram’s clinical utility was assessed using the DCA curve. All statistical analyses were performed using SPSS (version 25.0) and R software (version 4.2.1).

## Results

### Patient characteristics

A total of 66241 patients diagnosed with HCC from 2004 to 2015 were screened from the SEER database. After eliminating 64808 patients based on the exclusion criteria, a cohort of 1433 HCC patients with EBRT were included for further analysis. These patients were randomized 7:3 into a training cohort (n=1004) and an internal validation cohort (n=429). A total of 95 HCC patients with EBRT from the Department of Oncology, Third Xiangya Hospital, Central South University, Changsha, China, were included in an external validation cohort. The median OS for the whole SEER dataset and the external validation cohort was 10.0 and 14.1 months, respectively. The cumulative 1- and 2- year OS rates for the entire SEER dataset were 46.0% and 26.0%, respectively. In comparison, the cumulative 1- and 2- year OS rates for the external validation cohort were 64.2% and 25.3%, respectively. The baseline clinical, pathological, and other features of the training cohort, internal validation cohort, and external validation cohort are summarized in **Table 1**.

**Table 1:** The baseline clinical characteristics of the HCC in training cohort, internal validation cohort, and external validation cohort.

Variable	Training cohort (n=1004) (%)	Internal validation cohort (n=429) (%)	External validation cohort (n=95) (%)
Age (year)			
≤60	550 (54.8)	244 (56.9)	58 (61.1)
>60	454 (45.2)	185 (43.1)	37 b (38.9)
Sex			
Male	809 (80.6)	353 (82.3)	79 (83.2)
Female	195 (19.4)	76 (17.7)	16 (16.8)
Race			
White	734 (73.1)	311 (72.5)	NA
Black	136 (13.5)	65 (15.2)	NA
Others	134 (13.4)	53 (12.3)	95 (100.0)
Grade			
Well/Moderate	260 (25.9)	104 (24.2)	23 (24.0)
Poor/Undifferentiated	102 b (10.2)	39 (9.1)	8 (8.4)
Unknow	642 (63.9)	286 (66.7)	64 (67.6)
T stage			
T1/T2	618 (61.6)	265 (61.8)	52 (54.7)
T3/T4	386 (38.4)	164 (38.2)	43 (45.3)
N stage			
N0	870 (86.7)	374 (87.2)	81 (85.3)
N1	134 (13.3)	55 (12.8)	14 (14.7)
M stage			
M0	551 (54.9)	243 (56.6)	55 (57.9)
M1	453 (45.1)	186 (43.4)	40 (42.1)
Surgery			
No	896 (89.2)	369 (86.0)	82 (86.3)
Yes	108 (10.8)	60 (14.0)	13 (13.7)
Chemotherapy			
No	496 (49.4)	224 (52.2)	81 (85.3)
Yes	508 (50.6)	205 (47.8)	14 (14.7)
AFP			
Negative	152 (15.1)	56 (13.1)	48 (50.5)
Positive	422 (42.0)	168 (39.1)	44 (46.3)
Unknow	430 (42.9)	205 (47.8)	3 (3.2)
Size (cm)			
≤5	483 (48.1)	217 (50.6)	38 (40.0)
5-10	362 (36.1)	142 (33.1)	36 (37.9)
>10	159 (15.8)	70 (16.3)	21 (22.1)

NA, unavailable.

### Univariate and multivariate analyses

**Figure 2:**
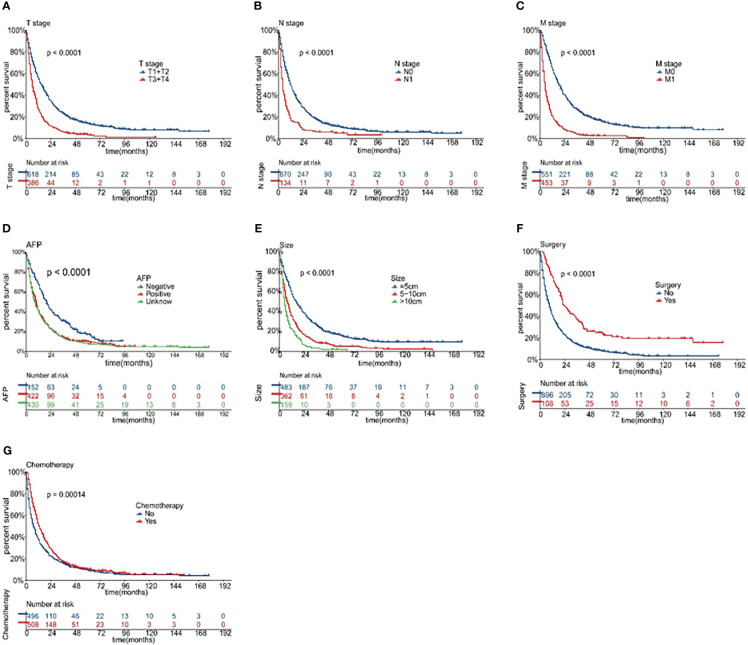
The Kaplan-Meier survival analysis curves for OS rates according to various independent risk factors: T stage **(A)**, N stage **(B)**, M stage **(C)**, AFP **(D)**, tumor size **(E)**, surgery **(F)**, and chemotherapy **(G)**.

Univariate and multivariate Cox regression analyses were performed on the training cohort to evaluate each prognostic factor (**Table 2**), T stage, N stage, M stage, AFP, race, grade, tumor size, surgery, and chemotherapy were significantly (P<0.05) identified in univariate analysis in the training cohort. the further multivariate regression analysis showed that T stage (P<0.001), N stage (P<0.01), M stage (P<0.001), AFP (P<0.01), tumor size (P<0.01), surgery (P<0.001), and chemotherapy (P<0.001) were independent prognostic factors for OS (**Figure 2**), which were included in the nomogram.

**Table 2:** Univariate and multivariate analyses of the clinicopathological parameters using the SEER training cohort.

Variable	Univariate			Multivariate			
	HR	95%CI	P value	HR	95%CI		P value
Age (year)							
≤60	1						
>60	0.99	0.87-1.13	0.908				
Sex							
Male	1						
Female	0.93	0.79-1.1	0.383				
Race							
White	1				1		
Black	1.26	1.04-1.52	0.019		1.14	0.94 - 1.38	0.1902
Others	1.04	0.86-1.27	0.688		0.91	0.75 - 1.11	0.3552
Grade							
Well/Moderate	1				1		
Poor/Undifferentiated	1.38	1.09-1.76	0.008		1.04	0.81 - 1.34	0.7673
Unknow	1.21	1.04-1.41	0.013		1.08	0.92 - 1.27	0.3217
T stage							
T1/T2	1				1		
T3/T4	1.99	1.74-2.28	P<0.0001		1.33	1.13 - 1.57	0.0008
N stage							
N0	1				1		
N1	2.03	1.68-2.45	P<0.0001		1.39	1.13 - 1.70	0.0016
M stage							
M0	1				1		
M1	2.94	2.57-3.37	P<0.0001		2.33	2.0 - 2.72	P<0.0001
Surgery							
No	1				1		
Yes	0.47	0.38-0.59	P<0.0001		0.57	0.45 - 0.71	P<0.0001
Chemotherapy							
No	1				1		
Yes	0.78	0.68-0.88	P<0.001		0.59	0.51 - 0.67	P<0.0001
AFP							
Negative	1				1		
Positive	1.61	1.31-1.97	P<0.0001		1.37	1.12 - 1.69	0.0026
Unknow	1.66	1.35-2.03	P<0.0001		1.39	1.13 - 1.70	0.0018
Size (cm)							
≤5	1				1		
5-10	1.79	1.55-2.07	P<0.0001		1.30	1.09 - 1.53	0.0026
>10	2.68	2.21-3.24	P<0.0001		1.55	1.24 - 1.93	0.0001

### Development and validation of prognostic nomogram for OS

**Figure 3:**
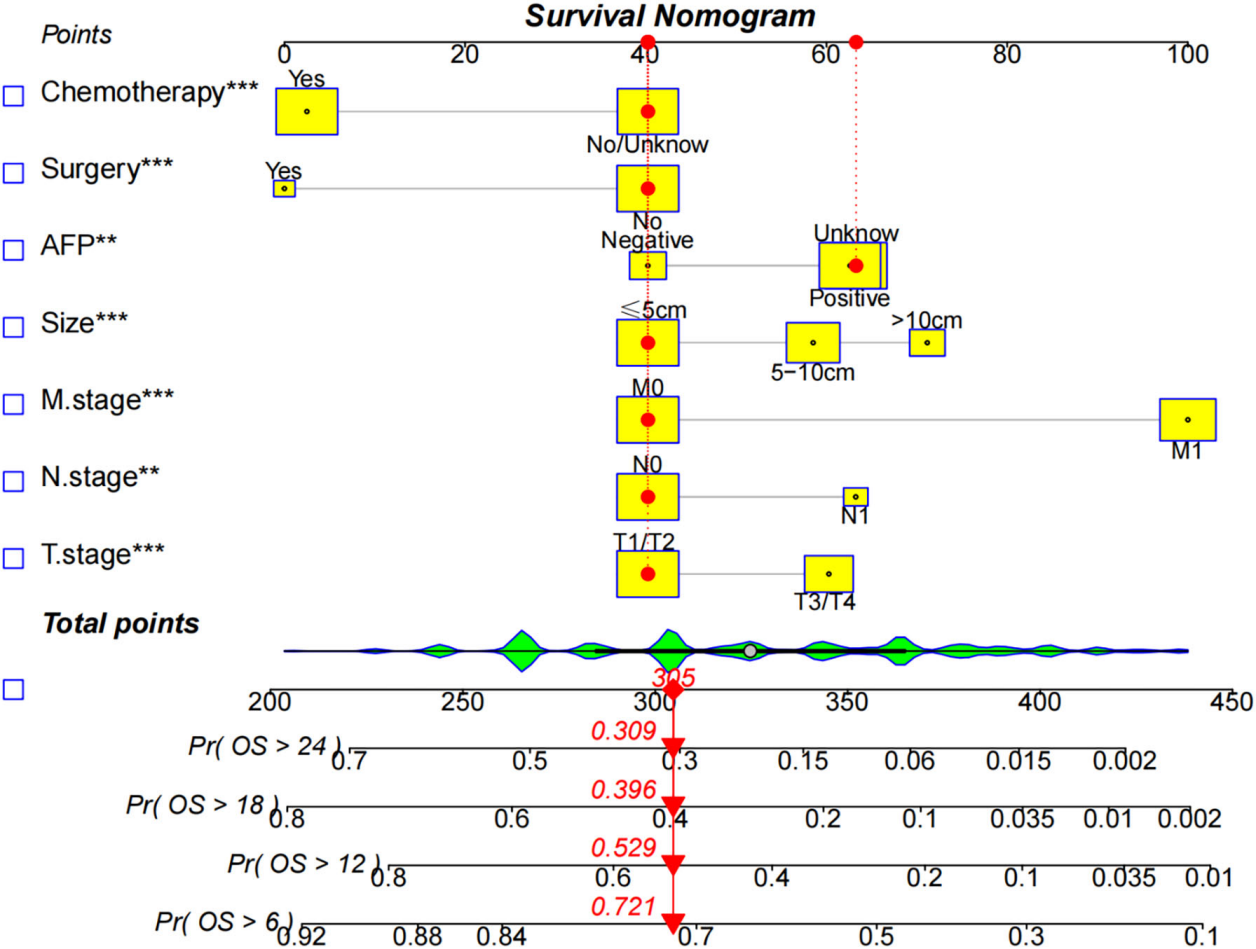
The nomogram predicts the 6-,12-,18-, and 24- month OS rates in HCC patients with EBRT. Give each factor a point based on the nomogram, the total points were obtained by adding the given points of all factors, the estimated 6-,12-,18-, and 24-months probabilities of OS of the individual patient can be easily obtained from the nomogram based on the total points.

A nomogram based on the selected prognostic factors from the training cohort was developed for predicting 6-, 12-,18- and 24- months OS in HCC patients undergoing EBRT (**Figure 3**). The nomogram demonstrated that the M stage contributed the most to prognosis, followed by surgery, chemotherapy, tumor size, N stage, AFP level, and T stage. Each level of every variable was assigned a score on the points scale; the total score was obtained by adding the scores for each selected variable, and predictions corresponding to this total score helped estimate 6-, 12-,18- and 24-months OS for HCC patients with EBRT.

**Figure 4:**
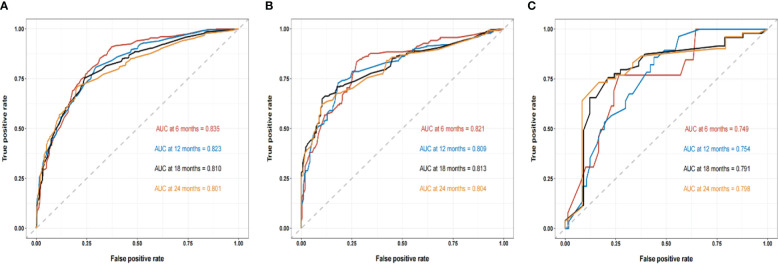
The ROC curves of the nomogram to predict 6-, 12-,18- and 24- months OS using the training cohort **(A)**, the internal validation cohort **(B)**, and the external cohort **(C)**, respectively.

**Figure 5:**
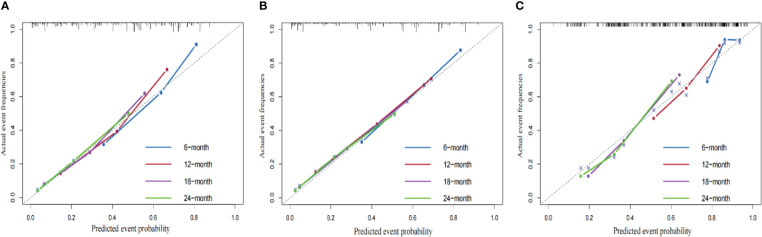
The calibration curves of the nomograms using three cohorts show how survival predictions from the model compare to the actual observed survival; the calibration curve of 6-, 12-,18- and 24- months OS for the training cohort **(A)**, internal validation cohort **(B)**, external validation cohort **(C)**.

**Figure 6:**
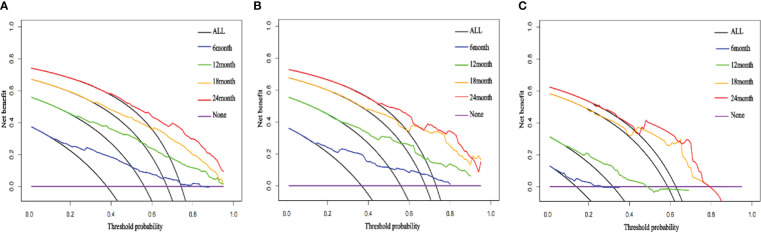
The DCA curves of the nomogram; the DCA curve of 6-, 12-,18- and 24- months OS for the training cohort **(A)**, internal validation cohort **(B)**, external validation cohort **(C)**.

We compared the AUC of each cohort (**Figure 4**). For the SEER training cohort, the AUC of predicting the 6-, 12-,18- and 24- months OS were 0.835 、0.823 、0.810 and 0.801, respectively. For the SEER internal validation cohort, the AUC for 6-, 12-,18- and 24- months OS were 0.821 、0.809 、0.813 and 0.804, respectively. For the external validation cohort, the AUC for 6-, 12-,18- and 24- months OS were 0.749 、0.754 、0.791 and 0.798 respectively. The C-index of the training cohort, internal validation cohort, and external validation cohort were 0.728 (95% confidence interval (CI): 0.716-0.740), 0.725 (95 CI%:0.701-0.750), and 0.696 (95 CI%:0.629-0.763), respectively; indicating a satisfactory discriminatory ability. Furthermore, calibration curves of each cohort were created for 6-, 12-,18- and 24-months OS and showed good consistency between nomogram prediction and actual observation (**Figure 5**).Finally, the DCA curves of the three cohorts show that this nomogram has good clinical utility (**Figure 6**). So our nomogram exhibited excellent predictive ability for HCC patients with EBRT.

### A web-based nomogram

**Figure 7:**
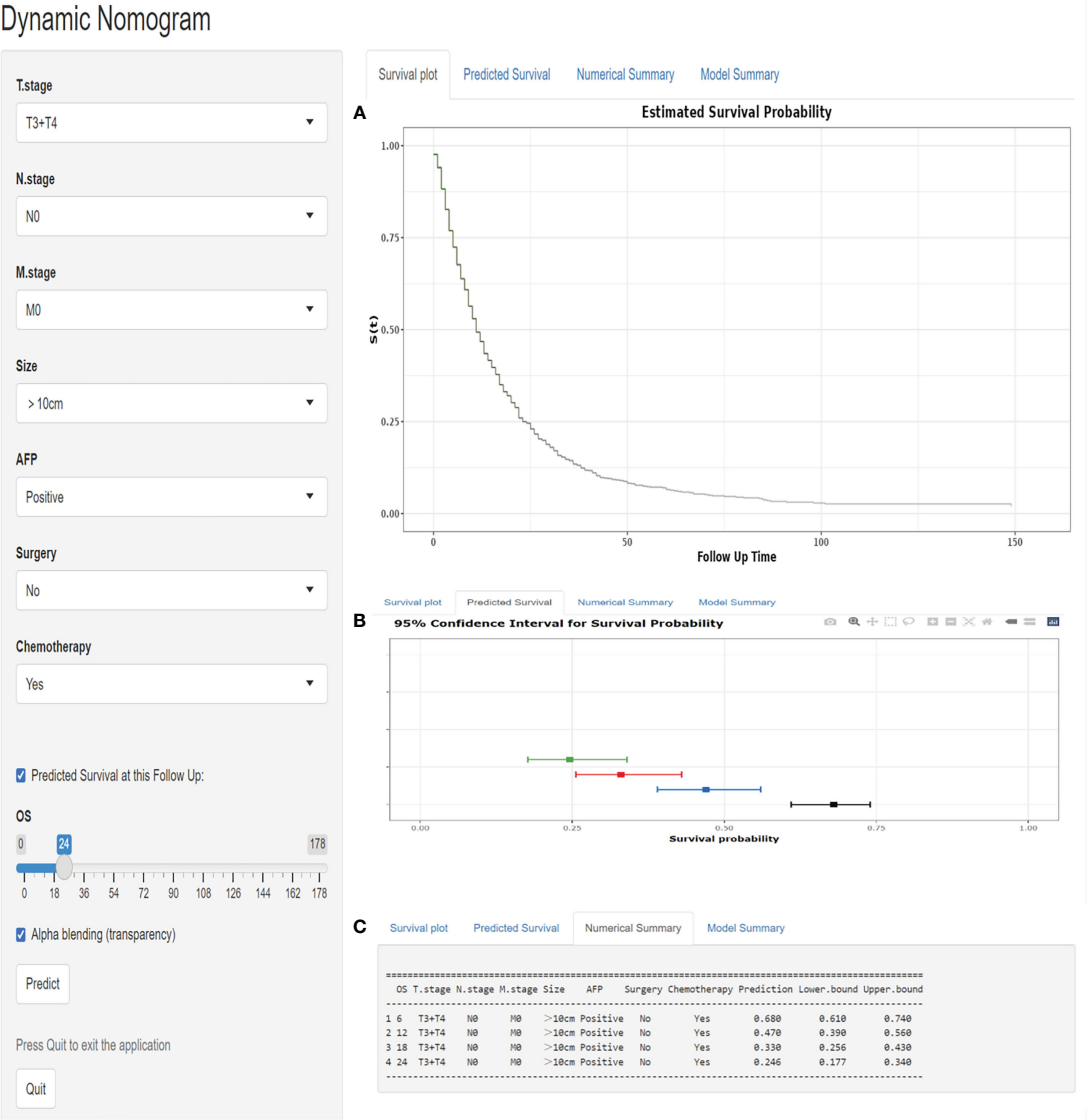
A web-based nomogram for predicting overall survival after EBRT with HCC patients. **(A)**The curve of estimated survival probability for those patients over time. **(B)** The 95% CI of the 6-, 12-, 18-, and 24- month survival probabilities for those patients. **(C)**The numerical summary of the 6-, 12-, 18-, and 24- month survival probabilities for those patients.

As seen in **Figure 7**, we designed a web-based nomogram for predicting overall survival in those patients, allowing clinicians and HCC patients to select clinical variables to visualize and personalize the prediction of survival probability after receiving EBRT. For example, we included an inoperable HCC patient with a tumor size of 140mm, a positive serum AFP value, and an AJCC stage of T3N0M0. After undergoing EBRT and chemotherapy, the estimated probability of survival for this patient at 6-, 12-, 18-, and 24- months was 68.0% (61.0-74.0%), 47.0% (39.0-56.0%), 33.0% (25.6-43.0%) and 24.6% (17.7-34.0%), respectively.

(https://zhangouling.shinyapps.io/HCC-with-EBRT-DynNomapp/ )

## Discussion

Global HCC incidence and mortality continue to rise (15). Due to the insidious onset and rapid progress of HCC, most of them are already advanced at the time of diagnosis; therefore, early local treatment methods such as liver resection, transplantation, and radiofrequency ablation have limited benefits (3–6, 16). With the continuous improvement of EBRT technology, its role has gradually changed from simple palliative treatment to multidisciplinary comprehensive treatment or even radical treatment (8–10, 17–21).

There is currently few complete and valid nomogram model to predict the prognosis of HCC patients treated with EBRT (12–14). Based on the sample data obtained from the SEER database, through univariate and multivariate analysis, a total of seven independent risk factors associated with prognosis were included: T stage, N stage, M stage, AFP status, tumor size, surgery, and chemotherapy; an intuitive prognostic prediction model was constructed, internal and external validation cohorts verified its accuracy. In addition, we designed a web-based nomogram to predict overall survival in those patients, which is expected to provide more evidence for individualized treatment.

It is well known that AJCC (TNM) staging is an important factor affecting the OS of HCC patients and has significant guiding value for its treatment (22). T stage has always been regarded as an important prognostic factor affecting HCC; it has been widely used in traditional HCC staging systems to provide treatment guidance, such as Okuda and BCLC stage (22–24). An Austrian study confirms that tumor cells in lymph nodes can spread into the vascular circulation and metastasize to distant organs (25); meanwhile, the National Comprehensive Cancer Network (NCCN) guidelines and the AJCC (TNM) staging system consider regional lymph node metastases as advanced HCC (26). The 5-year survival rate of patients with early-stage HCC after liver resection exceeds 70%, but once distant metastasis occurs, the median survival time is only 1-1.5 years even after multidisciplinary diagnosis and treatment (27, 28). Similar to previous studies, the T stage, N stage, and M stage in this study were all poor prognostic factors; it can be seen from the nomogram that the occurrence of distant metastasis is the most important factor affecting prognosis.

The serum AFP levels have been the most common laboratory value of HCC for decades. Liu et al. demonstrated that AFP levels are closely related to the degree of differentiation and vascular invasion of HCC (29, 30). In this study, HCC patients with negative AFP levels had lower scores and better prognoses, whereas HCC patients with unknown AFP levels had higher scores and poorer prognoses, probably because this part mainly consisted of patients with positive AFP levels. Tumor size is closely related to the prognosis of HCC, and many guidelines regard tumor size as an important reference for HCC staging and treatment (31). A multicenter study of surgical resection HCC demonstrated that tumors larger than 5cm had a worse prognosis than patients less than or equal to 5cm (32, 33). It is not difficult to see from the nomogram that as the tumor size increases, the higher the score and the worse the prognosis.

Surgery and chemotherapy were independent protective factors in this study. Surgical resection remains the preferred treatment for HCC; however, most HCC patients are asymptomatic in the early stage and are diagnosed in the advanced stage without the opportunity for surgery (6). Our current guidelines do not recommend surgery for advanced HCC (34). However, a retrospective study by Mao et al. showed that even with distant metastases, patients who underwent surgery when disease permitted had better outcomes than those who did not (35). Studies have shown that the combination of surgery and chemotherapy is beneficial for HCC patients (36, 37). Meanwhile, systemic chemotherapy with gemcitabine, doxorubicin or combined regimens also improved HCC patients survival (38). Similar to previous studies, less than 15% of patients in this study were operable. Still, it played a crucial role in the prognosis of HCC patients who received external beam radiation therapy. Likewise, the scores on the nomogram showed that patients who received chemotherapy had a better prognosis than those who did not.

The differences in the prognosis of HCC treated with EBRT among different ethnic groups may be related to the complex socioeconomic factors among ethnic groups and the differences in medical level in residential areas (39). A less differentiated tumor usually indicates a higher degree of malignancy, greater invasiveness, and a worse prognosis (40). There were differences in race and degree of differentiation in univariate analysis in this study, but no difference in multivariate analysis, which may be related to the small number of cases and the unknown degree of differentiation of most HCC patients, which needs further verification.

This study provides sufficient samples and clinical data based on the SEER database; a prognostic prediction nomogram was built and internal validation using eligible patients from the SEER database. Meanwhile, the external validation cohort from China was used for external verification, so the results have high reliability. The overall baseline characteristics of patients in the SEER database and Chinese patients were compared, all the Chinese patients were of the yellow race and the years of diagnosis were after 2014, and there were some differences in the treatment methods. However, The C-index, AUC values, calibration curves of the three cohorts all showed satisfactory results. The DCA curves suggested that the nomogram has good clinical utility. In addition, for the first time, we established a web-based, user-friendly nomogram model that clinicians and patients accessible from any electronic device.

Although the nomogram has good clinical utility, the present study had several limitations. First, data regarding several potential crucial prognosis-related serum markers such as HBsAg, AST, and CEA were unavailable in the SEER database; these will be the main part of our future research. Second, given the international and retrospective nature of the study, we cannot rule out that some clinicopathological characteristics might not have been evaluated uniformly in different institutions. In addition, there might be some selection bias in diagnosis, therapeutic strategies, and follow-up of patients across the institutions; variations in follow-up HCC patient condition changes and treatment plans might result in discrepancies in EBRT outcomes. Finally, compared with the American population-based cohort, the sample size of the external validation cohort from China was small; the validity of our nomogram in Eastern countries needs to be further evaluated.

## Conclusion

In conclusion, for the first time, we developed and validated a nomogram to predict overall survival in HCC patients with EBRT; both internal and external validation demonstrated remarkable calibration and discrimination of our nomogram. In addition, our established a web-based nomogram model can help clinicians judge prognosis, make better clinical decisions, and improve individualized survival probability.

## Data availability statement

The original contributions presented in the study are included in the article/**Supplementary Material**. Further inquiries can be directed to the corresponding author.

## Ethics statement

The studies involving human participants were reviewed and approved by the ethics committee at Third Xiangya Hospital, Central South University, Changsha, China. Written informed consent from the patients/participants was not required to participate in this study in accordance with the national legislation and the institutional requirements.

## Author contributions

PC designed the research. GZ and HP performed the research and analyzed results. GZ and LZ edited the manuscript. XX provided critical comments and revised the manuscript. YH, XW and ZD collected and organized data. LJ wrote the revised manuscript. All authors contributed to the article and approved the submitted version.
